# Segmental Thoracic Spinal Anesthesia for Critical Patients Undergoing Abdominal Surgeries: A Case Series and Literature Review

**DOI:** 10.7759/cureus.74348

**Published:** 2024-11-24

**Authors:** Yahya M Aljuba, Amro T Alkadi, Majde G Hamamdh

**Affiliations:** 1 Department of Anesthesia and Critical Care, Al-Ahli Hospital, Hebron, PSE; 2 Department of Technical Anesthesia, Al-Quds University, Jerusalem, PSE

**Keywords:** high-risk surgical patients, laparoscopic cholecystectomy, laparotomy, segmental thoracic spinal anesthesia, spinal anesthesia

## Abstract

Segmental thoracic spinal anesthesia (STSA) is emerging as a promising alternative for high-risk patients undergoing abdominal surgeries, particularly those who are not optimal candidates for general anesthesia (GA). By selectively targeting the thoracic spinal segments responsible for abdominal innervation, STSA aims to provide precise anesthesia and pain management while minimizing systemic side effects. This case series presents the outcomes of several critical patients who underwent abdominal surgeries under STSA. All patients who were considered at high risk for GA and underwent abdominal surgeries under STSA from January to June 2023 were involved in this study. Data regarding patient characteristics, surgical procedures, anesthetic outcomes, complications, and postoperative recovery were extracted and analyzed. The case series includes four patients, three of them underwent laparoscopic cholecystectomy and one underwent laparotomy. The results consistently demonstrated that STSA provided effective surgical anesthesia and muscle relaxation comparable to GA, with a better hemodynamic profile and a lower risk of systemic complications. Moreover, STSA exhibited a favorable postoperative recovery profile, including early ambulation, decreased opioid consumption, and improved patient satisfaction. The evidence suggests that STSA can enhance postoperative recovery and patient satisfaction. However, further research, including larger prospective studies and long-term follow-up, is warranted to establish its efficacy and safety more conclusively. STSA has the potential to become a valuable addition to the armamentarium of anesthesia techniques for abdominal surgeries, optimizing patient outcomes and improving the overall quality of care in this setting.

## Introduction

Abdominal surgeries often involve complex procedures and varying degrees of postoperative pain. While general endotracheal anesthesia has long been the standard approach for this kind of surgery, it is associated with potential risks, such as prolonged recovery time, systemic side effects, and complications related to airway management. In recent years, segmental thoracic spinal anesthesia (STSA) has gained recognition as a valuable alternative, revolutionizing the field of abdominal surgery anesthesia.

STSA involves a careful injection of local anesthetics at specific thoracic spinal levels, predominantly T7-T12, with a lower volume of local anesthetic than we used to inject in standard lumbar spinal anesthesia. By selectively blocking sensory and motor impulses originating from the abdominal organs and the anterior abdominal wall, STSA enables a highly targeted and precise form of anesthesia, limiting the spread of anesthesia to the areas essential for surgical intervention while preserving patient comfort and minimizing side effects.

The advantages of STSA extend beyond the avoidance of general anesthesia (GA)-related complications. In a study to compare GA to STSA for breast surgery, Paliwal et al. concluded that STSA provides better patient and surgeon satisfaction with significantly lower postoperative pain, longer time to first rescue analgesia, lesser postoperative opioid consumption, and lesser nausea and vomiting compared to GA [1].

Although STSA has many potential advantages, there are drawbacks as well. Major complications are rare and include direct trauma to the spinal cord by the spinal needle, infection, vertebral canal hematoma, spinal cord ischemia, total spinal anesthesia, or even death. Minor complications include hypotension, nausea/vomiting, bradycardia, paresthesia, transient mild hearing impairment, backache, urinary retention, and transient neurological symptoms. Lastly, post-dural puncture headaches, which are considered a “minor” complication, can be severely debilitating for patients and are common in occurrence [2].

The application of STSA requires meticulous planning, a thorough understanding of anatomical variations, and careful patient selection. Each case must be evaluated individually, taking into account factors such as surgical complexity, patient comorbidities, and anesthesia provider expertise. Additionally, a comprehensive knowledge of the relevant spinal anatomy, dosing considerations, and potential complications and their management is essential for successful implementation.

In this case series, we aim to comprehensively explore the role of STSA in abdominal surgeries. We conducted a literature review, focusing on clinical outcomes, safety profiles, patient satisfaction, and the impact on postoperative recovery.

By shedding light on the advantages, challenges, and potential risks associated with STSA, we hope to enhance patient care, improve surgical outcomes, and advance the field of anesthesia practice.

## Case presentation

In this case series, we are presenting four cases of STSA. Table 1 shows a brief overview of the anesthetics, procedure, and patient details.

**Table 1:** Brief description of the four cases presented in this case series. LA: local anesthetic; ASA: American Society of Anesthesiologists; M: male; F: female.

No.	Age/gender	ASA class	Surgery type	Level of LA injection	LA and adjuvants injected	Sensory block level	Surgery duration (minutes)
1	78/M	III	Laparoscopic cholecystectomy	T8-T9	Isobaric bupivacaine 7.5 mg + dexmedetomidine 5 mcg	T4-T12	58
2	70/M	III	Laparotomy, small bowel obstruction	T9-T10	Isobaric bupivacaine 7.5 mg + dexmedetomidine 10 mcg	T4-T12	64
3	65/F	III	Laparoscopic cholecystectomy	T8-T9	Isobaric bupivacaine 7.5 mg + dexmedetomidine 5 mcg	T4-L2	50
4	81/F	III	Laparoscopic cholecystectomy	T8-T9	Isobaric bupivacaine 7.5 mg + dexmedetomidine 5 mcg	T4-T12	72

It is important to note that all of these anesthetics took place in the morning hours (7:00 to 14:00) and not during night shifts, in the presence of at least one senior anesthesiologist, following discussion with the patient and the surgeon and after confirming that there would be a bed in the intensive care unit in case it was required. For each of the four patients, a backup GA plan was created in case the spinal block failed or there was a need to convert to GA for any reason. A previously published case report from the same institute was not included in this case series [3].

Case 1

Case 1 was a 78-year-old female, weighing 73 kg, with American Society of Anesthesiologists (ASA) class III, and a history of controlled hypertension, a previous transient ischemic attack, stage 4 chronic renal disease (CKD) with an estimated glomerular filtration rate (GFR) of 27.2 by modification of diet in renal disease (MDRD) equation, previous surgical history of two caesarian sections under GA, and a carotid endarterectomy two years ago. There was no history of specific cardiac or respiratory disease; however, the patient had a poor functional status with a metabolic equivalent of 3. The patient was admitted as a case of cholecystitis and scheduled for elective cholecystectomy. After evaluating the patient and discussing the case with the nephrology, surgery, and anesthesia teams, we concluded that the patient had a high risk for GA. We discussed the anesthetic options with the patient and explained the risks/benefits of GA and STSA; the decision was to proceed with STSA with a backup GA plan.

After obtaining an informed consent form, the patient was transferred to the operation room (OR), where 2 IV lines of 18G were secured, and 250 cc of lactated Ringer’s solution infused for pre-hydration. Standard monitors were applied, including electrocardiography (ECG), pulse oximetry, and non-invasive blood pressure (NIPB). The following baseline vital signs were obtained and recorded: heart rate (HR) = 84 beats per minute (BPM) and regular; oxygen saturation (SpO2) = 96% on room air (RA); and blood pressure (BP) = 150/84 mmHg.

**Figure 1:**
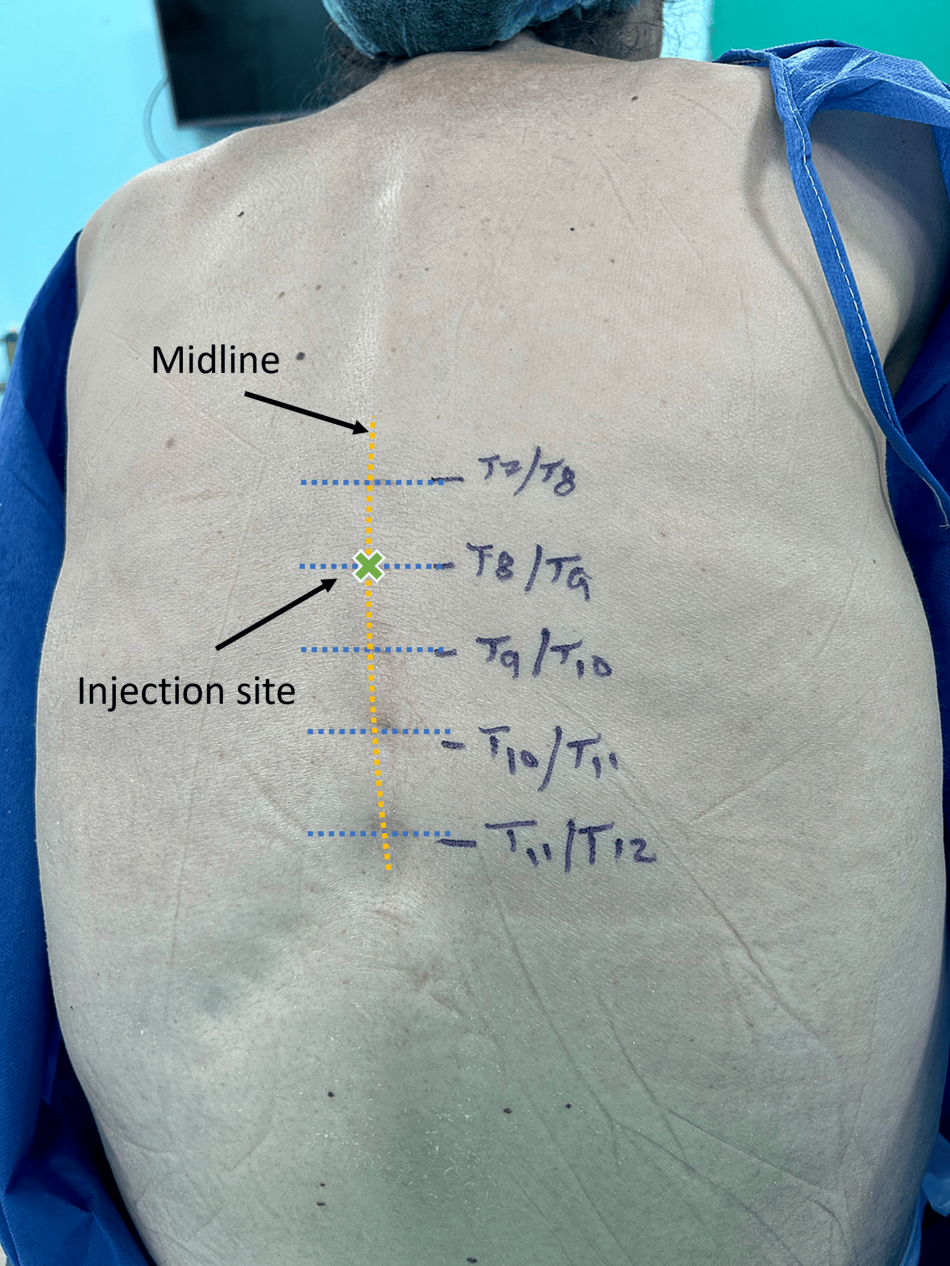
Determining the spinal interspace levels prior to injecting local anesthetic. The injection location is indicated by the X sign and imaginary lines are drawn for explanation.

A right radial artery line was placed following the infiltration of local anesthetic; then in the sitting position, the intervertebral levels were identified by palpation and marked (Figure 1), the skin was disinfected with alcohol-based chlorhexidine solution, and lidocaine 1% was infiltrated at the desired intervertebral space, then, using 25G pencil point spinal needle with an introducer (Pencan^TM^, B. Braun, Melsungen, Germany), the sub-arachnoid block was performed at T8-T9 interspace, by injecting isobaric bupivacaine (Bupivacaine-Grindeks 5 mg/mL, Grindex, Riga, Latvia) 7.5 mg (1.5 ml) with 5 mcg dexmedetomidine diluted in 0.5 ml (Precedex^TM^, Pfizer, New York, USA). The total volume injected was 2 ml. After the local anesthetic injection, the patient was returned to the supine position, and the sensory level was checked by the anesthesiologist using a pinprick test every one minute; after five minutes, the patient developed sensory anesthesia between T4 and T12 levels. Motor power was affected minimally, with a modified Bromage scale score of 1. Before allowing the surgery to start, the patient was urged to report any pain, discomfort, breathing difficulties, or other concerns, and the surgeon was requested to report any difficulties in performing the procedure due to anesthetic factors. Additionally, the patient was informed of the option to convert to GA if he experienced intolerable discomfort under STSA.

After confirmation of sensory loss level, surgery commenced with an intra-abdominal pressure limited to 12 cmH2O to minimize discomfort. The patient tolerated the pneumoperitoneum well and maintained spontaneous breathing with supplementary oxygen via a face mask at four liters per minute (L/M). From a hemodynamic prospect, the patient developed one episode of hypotension (defined as a drop in mean arterial pressure (MAP) of 20% or more), which responded well to boluses of ephedrine 5-10 mg and IV fluids (100-250 cc of lactated Ringer's solution). No episodes of bradycardia were noted (defined as HR < 50 BPM). The patient received ondansetron 4 mg and metoclopramide 10 mg as anti-emetic, paracetamol 1 gm IV as analgesics for postoperative analgesia, and dexamethasone 6 mg as an expert opinion to prolong the analgesic effect of spinal anesthesia.

The surgery lasted 58 minutes, and the surgical course was smooth and without significant complications. The reverse Trendelenburg position made it easier for the patient to breathe spontaneously. After completion of the surgery, the patient was kept in the postanesthesia care unit (PACU) for about 120 minutes to observe any possible early complications. Sensation in the targeted dermatomes was assessed regularly, sensory loss started to regress 86 minutes from the moment of local anesthetic injection, and within 90 minutes after the end of the surgery, there was a complete return of the sensation. The patient was then transferred to ICU to continue observation and to manage her postoperative pain. The patient remained pain-free until 128 minutes after the local anesthetic injection. Her visual analog scale (VAS) score was 2, 4, 4, and 3 out of 10 at two, four, six, and 12 hours, respectively. Postoperative analgesia was a challenge in this patient due to her CKD, and we opted not to perform epidural analgesia due to her CKD to avoid the risk of infection or bleeding. She received regular IV paracetamol, and a cautious dose of morphine sulfate 2 mg subcutaneously as needed by doctor's order. The patient needed only one dose of morphine rescue analgesia about seven hours after the surgery. On the next day, the patient had full motor and sensory function of lower limbs, she was fully mobile and out of bed, her pain was 2/4, and she returned to her usual state of health without considerable complications. She had no episodes of postoperative nausea and vomiting (PONV). The patient was discharged from the hospital on postoperative day three with a follow-up plan and contact details in case of emergencies.

Case 2

Case 2 was a 70-year-old male patient, 85 kg in weight, with a BMI of 31 and ASA class III, who was a known case of lung cancer status post left upper lobectomy seven months ago with a regular follow-up with his thoracic surgeon, and was on chemotherapy. The patient presented to the emergency department with acute abdominal pain and vomiting for several hours. After examination, a diagnosis of intestinal obstruction was made and the patient needed an urgent laparotomy to relieve the obstruction. A multidisciplinary discussion by anesthesia, surgery, and the patient’s thoracic surgeon (by phone) was made, and it was concluded that GA carries a high risk of complications due to his limited respiratory function and site of the surgery (upper abdominal surgery), and the plan was to proceed with the surgery under combined STSA-epidural anesthesia (STSA with epidural catheter insertion) to overcome any unintentional prolongation of the surgery, and to allow better postoperative pain management via epidural analgesia. It was made clear that if the patient does not tolerate the pneumoperitoneum, the procedure will convert to an open technique. After discussion with the patient and clarification of the risk-benefit ratio of general vs. regional anesthesia, the patient agreed to undergo the surgery under combined STSA-epidural anesthesia. A high-risk consent form was obtained and a postoperative ICU place was arranged. The patient was prepared, given antibiotics and IV fluids, and was transferred to the OR.

In the OR, standard monitors were applied (ECG, pulse oximeter, and NIBP). Baseline vital signs were taken and recorded and were as follows: BP = 112/64 mmHg; HR = 90 BPM regular; SPO2 = 94% on room air. After lidocaine infiltration, a left radial arterial line was inserted for invasive monitoring of the patient’s BP. Then, in the sitting position and with a full sterile technique, the T9-T10 interspace was detected, and lidocaine 1% was infiltrated. A Tuohy needle was cautiously advanced in median approach, loss of resistance to air was used, and after positioning the Tuohy needle’s tip in the epidural space (4 cm from the skin), a 27 pencil point spinal needle (Espocan combined spinal and epidural anesthesia tray, B. Braun) was advanced slowly and carefully to intrathecal space, after which free drainage of CSF was obtained, isobaric bupivacaine 0.5% (B. Braun) 7.5 mg (1.5 ml) and dexmedetomidine (Precedex^TM^, Pfizer) 5 mcg diluted in 0.5 ml were injected and the spinal needle was withdrawn (total volume of the injection was 2 ml). Then, the epidural catheter was gently advanced until 4 cm of the catheter was inside the epidural space, then the Tuohy needle was withdrawn, the epidural catheter was secured in space, and the patient was returned to the supine position. No drugs were injected into the epidural catheter so far. Assessment of sensory block was done every minute by the anesthesiologist using a pinprick test, and after five minutes, the patient developed a complete sensory block between T4 and T12, and motor function of the lower limbs remained grossly intact (with a modified Bromage scale of 2). After performing STSA, the patient received ondansetron 6 mg and metoclopramide 10 mg IV as anti-emetics, and dexamethasone 6 mg IV to extend the duration of the spinal block. After ensuring that the patient was hemodynamically stable and had a surgical sensory block, the surgery was allowed to start, and the patient remained on spontaneous breathing with supplementary oxygen via face mask at 4 L/M with a target SpO2 of > 94%.

The surgery was laparoscopic and the operative course was smooth. The surgery lasted 64 minutes, and the patient tolerated the procedure well without any pain or discomfort. From a hemodynamic point of view, the patient’s vitals remained stable during the surgery without any episode of hypotension, bradycardia, or desaturation. Estimated blood loss was about 150 ml, and urine output was about 200 ml. After completion of surgery, the patient was transferred to PACU for observation and assessment of pain and lower limbs' motor power. The sensory block started to regress after about 96 minutes of spinal injection. The patient was observed in the PACU for two hours and then transferred to the ICU for overnight observation. After ICU admission, the patient started to complain of postoperative pain, so an epidural test of 40 mg lidocaine 2% and 15 mcg of epinephrine were injected. After confirming a negative response to the epidural test dose, ropivacaine 2% with fentanyl 2 mcg/ml infusion was started, together with paracetamol 1 gm and ketorolac 30 mg IV as needed (PRN). VAS score was as follows: 1, 2, 3, and 2 out of 10 at two, four, six, and 12 hours, respectively. No episodes of PONV were recorded. On the next day, the patient was feeling well and returned to his baseline status, mobile and out of bed, and the pain was well tolerated. He was discharged on postoperative day two in good general condition with a follow-up plan and contact details.

Case 3

Case 3 was a 65-year-old female patient, 65 kg in weight, with a BMI of 23.6 and ASA class III, who was a known case of interstitial lung disease (fibrotic hypersensitivity pneumonitis) on regular treatment with prednisolone and home oxygen therapy 2 L/M for several hours per day. The patient presented to the ER with recurrent right upper quadrant (RUQ) abdominal pain. She was diagnosed with acute calculous cholecystitis and planned for laparoscopic cholecystectomy.

The patient had a history of moderate exercise intolerance, with an average O2 saturation of 88-90% on room air at rest, while after exercise, her saturation may fall to 70s if she was not treated by oxygen. On examination at the emergency department, the patient was conscious and oriented, in moderate abdominal pain, and her vitals were as follows: BP = 144/81 mmHg; HR = 83 BPM regular; SpO2 = 90% on room air; respiratory rate (RR) = 16 cycles per minute (CPM). Unfortunately, arterial blood gas (ABG) and imaging studies of the patient were lost due to a computer software issue, and could not be restored. A multidisciplinary discussion by anesthesia, surgery, and pulmonologist concluded that the patient was risky for general endotracheal anesthesia and the plan was to do STSA. After a discussion with the patient about the risk/benefit ratio of GA and STSA, she agreed the surgery to be done under STSA with a backup GA plan. After obtaining an informed consent form, the patient was prepared and transferred to the OR.

**Figure 2:**
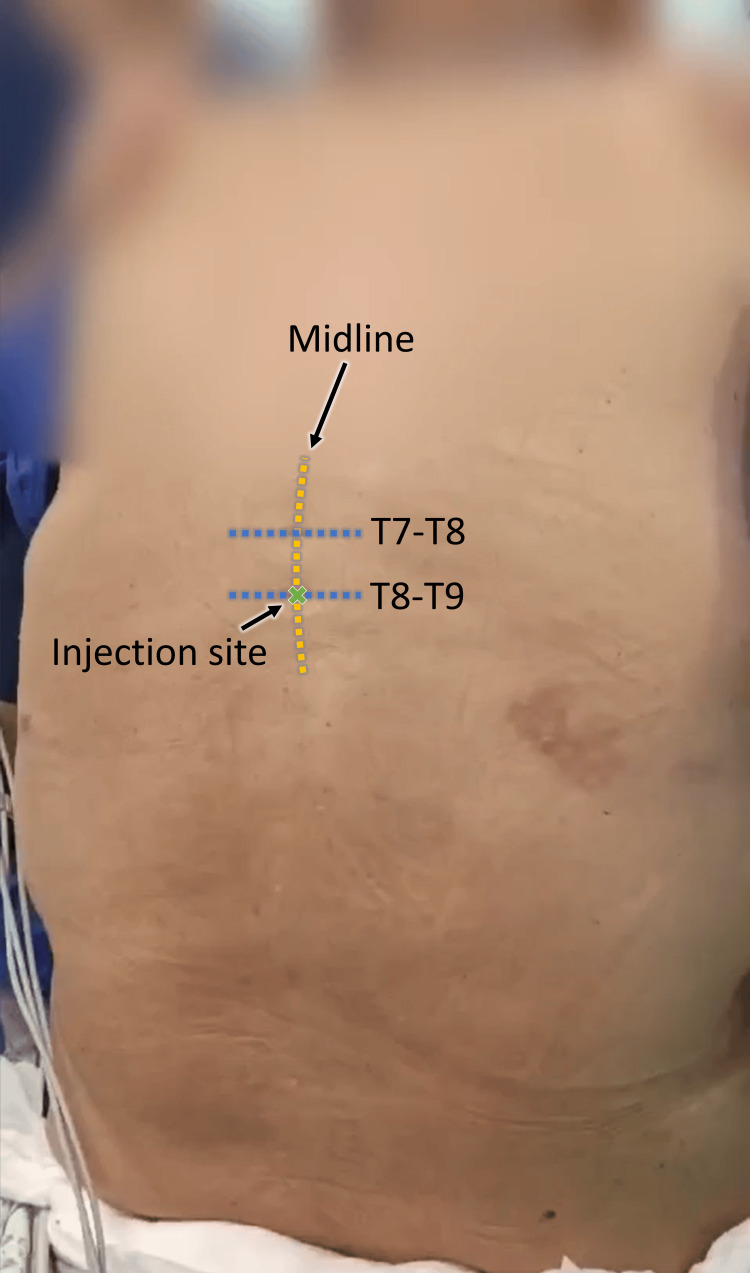
Determining T8-T9 spinal interspace levels prior to injecting local anesthetic. The injection location is indicated by the X sign and imaginary lines are drawn for explanation.

In the OR, standard monitors (ECG, pulse oximeter, and NIBP) were applied. Pre-induction vital signs were as follows: BP = 140/75 mmHg; pulse = 79 BPM and regular; temperature = 36.6°C; RR: 15 CPM; SpO2 = 89% on room air. After that, a right radial arterial line was inserted after local anesthetic infiltration. Two wide-bore (18G) IV cannulas were applied and 200 ml of lactated Ringer's solution was administered slowly while performing the procedure. Under complete sterile technique with the patient in the sitting position, the correct intervertebral space, located between T8 and T9, was identified (Figure 2) and the skin of the puncture site infiltrated with 2 ml of lidocaine 1%. The puncture was performed via a median approach using 25-gauge Pencan (B. Braun). Once a clear flow of CSF was established, isobaric bupivacaine 0.5% (B. Braun) 7.5 mg (1.5 ml) and dexmedetomidine (Precedex^TM^, Pfizer) 5 mcg diluted in 0.5 ml were injected, as shown in Video 1 (total volume of injection was 2 ml). The patient was then returned to the supine position with a 10° head-up position to aid her ventilation. The sensory level was checked every two minutes using a pinprick test. After five minutes, an adequate sensory block was confirmed between T5 and L2 levels via a pinprick test by the anesthesiologist, with a slight decrease in lower limbs' power with a modified Bromage scale score of 2 in both lower limbs. The patient was given ondansetron 6 mg, metoclopramide 10 mg, dexamethasone 6 mg, ketorolac 30 mg, and paracetamol infusion 1 gm. After confirming the anesthetic sensory block in the mentioned dermatomes, the surgery was allowed to start.

**Video 1:** Performing segmental thoracic spinal anesthesia at T8-T9 for laparoscopic cholecystectomy.

The surgery lasted about 50 minutes and the patient tolerated the procedure well and without any pain or discomfort. The patient was spontaneously breathing and received oxygen via a simple face mask with a flow of 5 L/M, and SpO2 was maintained at 95-98% all over the procedure. During the procedure, the patient was observed closely, and instructed to report any pain or discomfort immediately. There were no episodes of hypotension or bradycardia during the procedure. Sensory loss lasted about 110 minutes, after which a gradual return of sensation was noticed. A total of 500 ml of Ringer's lactate was infused slowly during the procedure. Postoperative analgesia was maintained with regular intravenous paracetamol and ketorolac. Postoperatively, the patient was admitted to the ICU for close observation, VAS scores were 1, 2, 2, and 3 out of 10 at two, four, six, and 12 hours postoperatively. There were no episodes of POVN. On the next day, the patient returned to her baseline physical status, with minimal pain, so she was transferred to the surgical floor for observation and management and was discharged on postoperative day four in her baseline physical status, with a follow-up plan and contact details.

Case 4

Case 4 was an 81-year-old woman, weighing 102 kg, with a BMI of 40.9 and ASA III classification. She had a medical history of obesity, controlled diabetes mellitus, hypertension, and hyperlipidemia, and a history of limited exercise tolerance due to her obesity (metabolic equivalent of 4). There was no history of cardiac disease and no prior surgical interventions. The patient presented to the emergency department with recurrent RUQ pain, and a diagnosis of acute calculous cholecystitis was made. The patient was then scheduled for laparoscopic cholecystectomy.

On examination at the surgical floor, the patient had the following vital signs: BP = 148/78 mmHg; HR = 74 BPM regular; RR = 17 cycles/minute; temperature = 36.8°C; O2 saturation = 94% on room air. Cardiac auscultation revealed normal S1 and S2, with no added sounds, and chest auscultation revealed mild bilateral wheezes. Given her obesity, advanced age, and limited exercise tolerance, a thorough discussion involving both surgical and anesthesia teams led to the decision to pursue STSA as a primary plan of anesthesia, with GA being a backup plan.

The plan of anesthesia was discussed with the patient, with a thorough explanation of the advantages and disadvantages of this approach, and it was explained clearly to the surgeon and the patient that if the STSA anesthesia was not satisfactory, a conversion to GA would be made. The patient agreed to undergo the procedure under STSA. An informed consent form was obtained, and the patient was prepared and transferred to the OR.

In the OR, a new 18 G intravenous cannula was applied (the total was two 18 G IV cannulas), and the patient received IV hydration with 250 ml Ringer’s lactate solution, after that ECG, pulse oximetry, and NIBP were monitored. Baseline vital signs were as follows: BP = 159/90 mmHg; HR = 65 BPM regular; RR = 18 CPM; O2 saturation = 95% on room air.

Subsequently, a left radial arterial line was inserted for invasive BP monitoring. After careful examination of the patient’s back, T8-T9 interspace was identified, complete sterilization by chlorhexidine was made, a sterile covering was applied, and lidocaine 1% was infiltrated as a local anesthesia. A 25 G pencil point spinal needle with an introducer was introduced cautiously. After about 1 cm from the skin, the introducer was fixed in place and only the spinal needle was allowed to continue slowly and cautiously with frequent check for CSF flow. When a free flow of CSF was observed, 7.5 mg of isobaric bupivacaine 0.5% (1.5 ml) with dexmedetomidine 5 mcg (0.5 ml) was injected (total volume of 2 ml), and the patient returned to the supine position with a head-up position of 20 degrees to relieve his shortness of breath.

After local anesthetic injection into the subarachnoid space, the sensory level was checked every minute via a pinprick test, and after six minutes, a block level of T4-T12 was confirmed by the anesthesiologist. The motor function was minimally affected with both lower limbs exhibiting a motor power of 2 on the modified Bromage scale. With confirmed sensory blockade, the surgery started smoothly without complication, maintaining a maximum intra-abdominal inflation pressure at 12 cmH2O to alleviate patient discomfort. Shoulder pain prompted the administration of IV paracetamol and Optalgin, and was effectively managed throughout the procedure, while the patient maintained spontaneous breathing with supplemental oxygen via face mask at 4 L/M.

The surgery lasted 80 minutes, with vigilant anesthesia monitoring and continuous patient communication. Post surgery, the patient was observed in the PACU for two hours, during which a gradual return of sensory function was started after approximately 120 minutes from the time of the subarachnoid injection.

**Figure 3:**
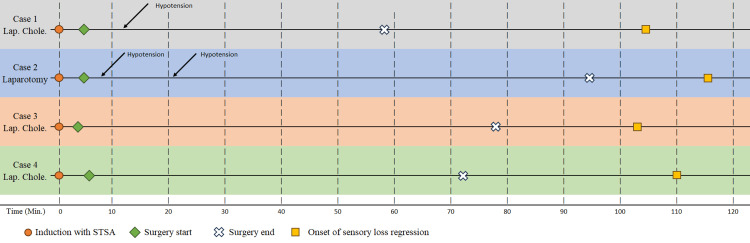
Illustration of the timeline of the four procedures. STSA: segmental thoracic spinal anesthesia.

The patient was found to be stable enough to be transferred to the surgical floor for continued monitoring. The postoperative pain management protocol consisted of paracetamol IV, regular ketorolac, and IV morphine PRN. The patient reported a pain score of 2/10 on the VAS scale after two hours, increasing to 3/10 after four hours, and 4/10 after six hours. Remarkably, by the following day, the patient demonstrated full mobility and sensation, she was discharged on postoperative day three with minimal complications, returning to her baseline health status. She was instructed regarding follow-up at the surgical clinic, and given a contact number to call in case of emergency (Figure 3).

## Discussion

Literature review

Historical Background

Spinal anesthesia and segmental spinal anesthesia are not wholly novel techniques; rather, German surgeon August Karl Gustav Bier (24 November 1861-12 March 1949) was the first to conduct both intravenous regional anesthesia (often referred to as Bier’s block) and spinal anesthesia. Spinal anesthesia was the first regional anesthetic technique, and the first spinal anesthesia procedure took place in Germany in 1898 [4,5].

When it comes to thoracic spinal anesthesia, Professor Thomas Jonnesco originally described the technique in Romania in 1909. Depending on the type of surgery needed, he defined two puncture locations, the T1-T2 and T12-L1 interspaces, and named his method "general spinal analgesia" [6,7].

However, “segmental spinal” was described for the first time only in 2006 by Van Zundert et al. He proposed a segmental spinal block for laparoscopic cholecystectomy in a patient with severe obstructive lung disease, utilizing a low thoracic puncture (T10) and combined spinal epidural approach [8].

Indications of Segmental Spinal Anesthesia

STSA is generally preserved for selected procedures and patient populations, typically for shorter procedures (less than two hours) with patients at high risk under GA. Operations that have been performed with success include abdominal cancer surgeries, breast cancer surgeries, cholecystectomies (open and laparoscopic), and open nephrectomy [2,9-16].

Firstly, it should be made clear that STSA should only be performed by highly experienced anesthesiologists who have extensive practice with neuraxial anesthesia, and only when there are sufficient monitoring and hospital resources (e.g., multi-disciplinary team discussion, a supportive environment, and an intensive care unit when needed).

It is worth mentioning that STSA should not be considered for long procedures, unless an intrathecal catheter for continuous spinal anesthesia is placed, or a combined spinal-epidural (CSE) technique is performed. After reviewing various resources, the duration of the motor block in STSA is short compared with the sensory block, and the surgical time ranges from 23 to 136 minutes with a single shot spinal block [6].

When considering STSA, it is critical to inform the patients that they will be conscious during the process and that they may experience some discomfort, such as tugging or pain from pneumoperitoneum from insufflation during a laparoscopic procedure.

Techniques and Positions

Before deciding to perform STSA, it is recommended to evaluate the vertebral column for possible anatomical variations, surgeries, infections, etc. Also, basic neurological exams and motor and sensory functions should be assessed and documented.

STSA can be done either in a sitting or lateral position; however, the risk of spinal cord injury during the thoracic level spinal needle insertion is reduced when the patient is seated head-down, as this position enhances the posterior separation of the dura mater and spinal cord.

When it comes to the level of intervertebral space, it is highly dependent on the surgery requested and the volume and type of LA injected, and generally can be performed from T4/T5 and up to T10-T11. Different approaches to the space, volume, and sensory levels of STSA are shown in Table 2 [6,10,11].

**Table 2:** Different approaches, levels, and sensory levels of STSA. * Some references mention performing spinal anesthesia at the level of T7-T8 for open nephrectomy [9,12]. STSA: segmental thoracic spinal anesthesia; LA: local anesthetic.

Surgery type	Intervertebral space of injection	Volume of LA injection	Sensory level obtained
Breast surgery [6,17]	T5-T6	1.4	T1-T11
Colon resection [6,18]	T4-T5	1.7	T3-T5
Gastrectomy [6,11]	T6-T8	1	Up to T4
Laparoscopic cholecystectomy [6,15,16]	T8-T10	1.5-2.5	1.5 ml LA: T3-L2; 2.5 ml LA: T2-L5
Nephrectomy* [6,9,10,12]	T10-T11	1.5-2	T6-T12

**Figure 4:**
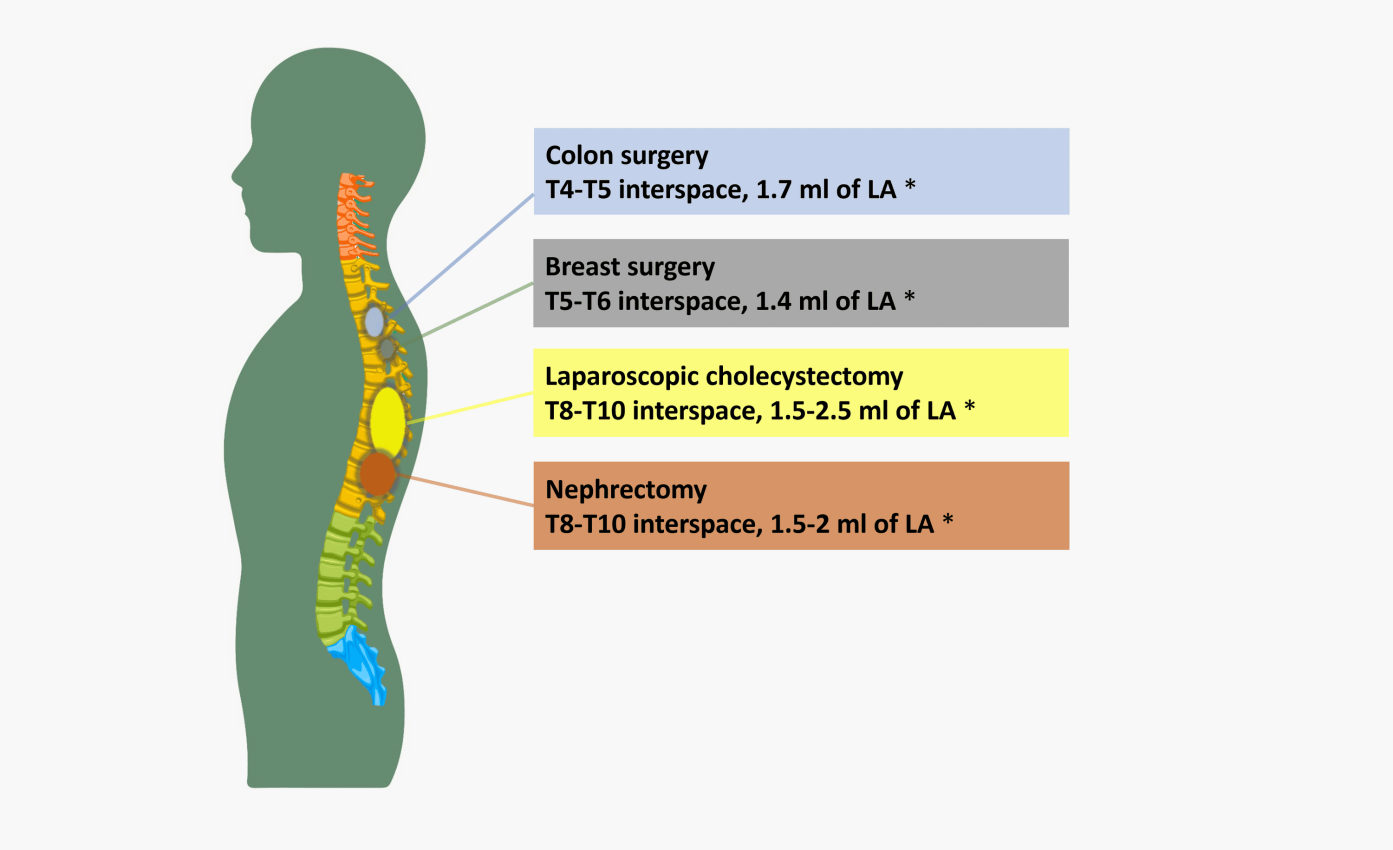
Schematic overview of the suggested level of local anesthetic injection in STSA for some surgeries. * See the explanation below regarding the type of local anesthetics used. This illustration was designed by the authors and inspired by a previous publication [6]. STSA: segmental thoracic spinal anesthesia; LA: local anesthetic.

For clarity, we have designed an illustration showing the level and volume of the local anesthetic injection for various surgeries under STSA (Figure 4).

Regarding the type of spinal needles to be used in STSA, the cutting-type needle, with a terminal orifice, appears to be a safer alternative. Both median and paramedian approaches are described in the literature. The median approach is described as technically more challenging in STSA compared with the paramedian approach for several reasons: epidural fat and vessels, ossification of the ligamentum flavum, and the sharply angled spinous processes from T4 to T9 pointing caudally [4,5].

The local anesthetics used for STSA are typically long-acting agents, such as levobupivacaine, bupivacaine, or ropivacaine with both hyperbaric and isobaric formulations. When considering the baricity of local anesthetics, hyperbaric solutions appear to have a greater impact on the posterior (sensory) roots compared to the anterior (motor) roots when the patient is in the supine position; this leads to a more noticeable sensory block than a motor block. The opposite is true for isobaric and hypobaric solutions, where the blockade of the anterior roots results in a stronger motor than sensory block [6].

Previous Studies

STSA has been used by many anesthesiologists from different regions of the world, and for various types of surgeries, with different outcomes and recommendations.

One of the largest studies on STSA is a study conducted by Chandra et al. in India, where the authors studied 2174 patients with ASA I and II class, who underwent laparoscopic cholecystectomy under thoracic spinal anesthesia. All the blocks were performed at the T10-T11 intervertebral space using a median approach utilizing a 26-gauze Quincke Babcock needle, and the block was successful in all the patients. The block was performed using hyperbaric bupivacaine 0.5% of 2.4 ml with 5 mcg of dexmedetomidine. The first attempt of dural puncture was successful in the majority of patients (92.0%). The average time for regression of the sensory block was 136 minutes. As per their results, spinal anesthesia was successful in 2,074 patients, and in 92% of cases, spinal anesthesia was successful from the first attempt. Of all the patients, 5.8% experienced paraesthesia during needle insertion. Regarding events after spinal anesthesia, hypotension was observed in 18% of patients, bradycardia in 13%, nausea in 10%, and shoulder pain in 6%. Most (94%) of the patients were very satisfied with the procedure. None of the patients experienced postoperative adverse effects [16].

This study highlights the potential of STSA in laparoscopic cholecystectomy procedures, being relatively easy to perform and generally a safe option. However, the study involved only ASA class I and II patients, which may lead to confusion when planning to perform STSA on ASA class III patients. In our experience, we used a dose of 1.5 ml of 0.5% (7.5 mg) isobaric bupivacaine, while the authors from India used a standard dose of 2.4 ml of 0.5% isobaric bupivacaine. This may lead to a slightly longer mean duration of sensory block (136 minutes), while in our experience, the sensory block regressed more rapidly. This may highlight the need to think about the nature and length of the surgery and to calculate the dose on the risk-benefit ratio.

Van Zundert et al. from the Netherlands conducted a study on 20 patients with ASA class I and II who underwent laparoscopic cholecystectomy under STSA. The authors used T10-T11 as a standard level of local anesthetic injection. However, they used a combined spinal-epidural technique, where epidural space was detected by loss of resistance to air, and then a 27-gauge pencil-point spinal needle was inserted gently inside the Tuohy needle. Once a clear flow of CSF had confirmed the correct placement of the spinal needle, 1 ml of plain bupivacaine 5 mg/ml mixed with 0.5 ml of sufentanil 5 mcg/ml was injected before the spinal needle was removed. The epidural catheter was then advanced into the epidural space, the Tuohy needle withdrawn, and the catheter taped in place, leaving 4 cm in the space. Motor (modified Bromage scale) and sensory (pinprick) blocks were tested after the block until a minimal block of T4-T12 sensory block was confirmed by pinprick. The intra-abdominal pressure was limited to 12 mmHg. Their results showed this technique to be successful in all 20 patients. Effective sensory block was obtained in 15 minutes or less in all patients, The cardiovascular changes were minimal, treated with crystalloids and ephedrine. The mean duration of the surgery was 60 (SD = 21) minutes and was completed 78 (SD = 20) minutes after spinal injection [8].

This study, which was also conducted on ASA class I and II patients, revealed the relatively safe and effective nature of STSA. However, no patient required top-up or boluses via the epidural catheter, which may question the need to insert the epidural catheter in the first place. The dose of local anesthetic used was relatively small (1 ml of 0.5% isobaric bupivacaine or 5 mg), and this dose was shown to be effective for surgical anesthesia, but the sensory block started to regress after a mean time of 75 minutes, which may be enough if the surgery was straight forward, but maybe considered inadequate in case of unexpectedly prolonged surgery. In this case, epidural anesthesia may be considered helpful.

Another study was done in Italy by Vincenzi et al. on nine patients with ASA class I, II, and III using hypobaric 0.25% 5 mg and isobaric ropivacaine 0.25% 10 mg. They reported high patient satisfaction and no need for conversion to GA in any patient. Their study concluded that STSA is a safe, reliable, and adequate anesthetic method in elective laparoscopic cholecystectomy, and represents a reasonable alternative to GA [11].

The primary limitation of our case series is the small number of patients, which restricts the generalizability of the findings. While the results are promising and demonstrate the potential benefits of STSA in critical patients undergoing abdominal surgeries, the limited sample size makes it difficult to draw definitive conclusions. Larger studies, including randomized controlled trials, are needed to validate these findings and assess the broader applicability and safety of this technique across diverse patient populations.

## Conclusions

Although GA is the standard approach for a variety of intra-abdominal surgeries, such as laparoscopic surgeries or laparotomies, there is growing evidence supporting the use of STSA for abdominal laparoscopic surgeries, especially in high-risk patients. However, careful planning and patient selection are mandatory. STSA provides reliable surgical anesthesia and is relatively safe, tolerable, and easy to perform. The risk-benefit calculation is mandatory when considering STSA. The expected duration of surgery, patient cooperation, tolerance, surgeon's comfort, and hospital set-up should all be considered.
